# Attentional Bias Modification Training for Itch: A Proof-of-Principle Study in Healthy Individuals

**DOI:** 10.3389/fmed.2021.627593

**Published:** 2021-06-30

**Authors:** Antoinette I. M. van Laarhoven, Jennifer M. Becker, Dimitri M. L. van Ryckeghem, Stefaan Van Damme, Geert Crombez, Reinout W. H. J. Wiers

**Affiliations:** ^1^Health, Medical, and Neuropsychology Unit, Faculty of Social and Behavioural Sciences, Leiden University, Leiden, Netherlands; ^2^Leiden Institute for Brain and Cognition, Leiden University, Leiden, Netherlands; ^3^Department of Experimental-Clinical and Health Psychology, Ghent University, Ghent, Belgium; ^4^Research Unit Integrative Research Unit on Social and Individual Development (INSIDE), Institute of Health and Behaviour, Faculty of Humanities and Social Sciences, University of Luxembourg, Esch-sur-Alzette, Luxembourg; ^5^Section Experimental Health Psychology, Clinical Psychological Science Departments, Faculty of Psychology and Neuroscience, Maastricht University, Maastricht, Netherlands; ^6^Centre for Pain Research, University of Bath, Bath, United Kingdom; ^7^Addiction Development and Psychopathology Laboratory, Department of Psychology, University of Amsterdam, Amsterdam, Netherlands

**Keywords:** itch, attention, attention bias, attention bias modification (ABM), attention training, pruritus, psychodermatology, dot-probe paradigm

## Abstract

Itch draws our attention to allow imposing action against bodily harm (e.g., remove insects). At the same time, itch is found to interfere with ongoing tasks and daily life goals. Despite the key role of attention in itch processing, interventions that train individuals to automatically disengage attention from itch cues are lacking. The present proof-of-principle attention bias modification (ABM) training study was aimed at investigating whether attention to itch as well as sensitivity to mild itch can be changed. Healthy volunteers were randomized over three ABM-training conditions. Training was done via a modified pictorial dot-probe task. In particular, participants were trained to look away from itch stimuli (*n* = 38), toward itch stimuli (*n* = 40) or not trained toward or away from itch at all (sham training, *n* = 38). The effects of the ABM-training were tested primarily on attention to itch pictures. Secondarily, it was investigated whether training effects generalized to alterations in attention to itch words and mechanical itch sensitivity. The ABM-training did not alter attention toward the itch pictures, and there was no moderation by baseline levels of attention bias for itch. Also, attention bias to the itch words and itch sensitivity were not affected by the ABM-training. This study was a first step toward trainings to change attention toward itch. Further research is warranted to optimize ABM-training methodology, for example increasing motivation of participants. Eventually, an optimized training could be used in patient populations who suffer most from distraction by their symptoms of itch.

**Clinical Trial Registration:** Identifier: NL6134 (NTR6273). The website URL is: https://www.trialregister.nl/

## Introduction

Itch, and particularly chronic itch interferes with one's behavior and psychosocial functioning (1–3). In turn, reduced psychosocial well-being can intensify itch, resulting in a vicious cycle (4, 5). Unique for itch compared to pain is that it can be further amplified or even induced audiovisually (e.g., by hearing scratch sounds or looking at pictures of scratching people); i.e., itch is contagious and this is amplified in itch patients (6). A key mechanism of contagious itch is attention (7, 8). Focusing attention on potential threat is essential to sort its nocifensive function and protect skin integrity (9, 10). Since attention may play a central role in the vicious circle of itch amplification (11) and psychological burden (12–16), interventions targeting attention to itch seem promising.

Research indicates that patients with chronic itch may have increased attention (AB; attention bias) toward words related to itch compared to neutral words (17), and compared with healthy controls (18). Similarly, healthy individuals display an AB toward itch words and pictures (12, 14), although evidence is equivocal (16). Some techniques have been investigated to reduce itch temporarily (19, 20), but no strategies exist that reduce AB to itch; hence attention strategies effectuating longer-term itch relief are lacking. Attention bias modification (ABM) training for itch may offer a solution, as such a training has been shown effective in other fields (21).

In the domain of pain, closely related to itch (9, 22), ABM-trainings have been developed to alter AB for pain. Such ABM-trainings aim to train individuals to automatically focus attention at neutral stimuli while concurrently displaying pain-stimuli (pain-related words and/or painful faces). Initial studies in patients with chronic pain indicated that single- as well as multi-session ABM-trainings could reduce pain sensitivity (23–25). In healthy individuals, ABM-trainings affected pain thresholds, pain tolerance, or experienced pain (26–29), and some studies demonstrated altered AB for pain (26, 27). In addition, a study has shown that effects of an ABM-training with words generalized to effects on AB for painful faces after the training (27). Furthermore, individual characteristics, like catastrophizing or the ability to inhibit attention to irrelevant information (as feature of executive control), may play a role in (the retraining of) AB for pain (30–32). All in all, evidence on ABM-training effectiveness in pain as well as the role of individual characteristics is equivocal (28, 29, 33–35). Overall, based on theory and promising evidence in pain, it seems worthwhile to investigate whether an ABM-training for itch would be effective to reduce itch sensitivity and/or AB toward itch. However, to our knowledge, an ABM-training for itch has not yet been developed. As a first step of intervention-development, it should be verified whether AB toward itch can be trained—in either direction (i.e., in a proof-of-principle study) (36).

In this proof-of-principle study, we aimed to investigate whether AB to itch pictures can be altered by an ABM-training away from and toward pictorial itch stimuli. Furthermore, we investigated whether these effects would generalize to altered AB to itch words and actual itch sensitivity. It was hypothesized that, when compared to sham training, an ABM-training away from itch pictures would result in AB away from itch pictures and words as well as a lowered itch sensitivity, whereas an ABM-training toward itch would effectuate the opposite. Additionally, the possible role of individual characteristics, including general attentional inhibition, neuroticism, and itch catastrophizing, in the ABM-training effects was explored. Moreover, given some recent evidence (37), we explored *post-hoc* whether the training effects were moderated by the baseline AB for itch.

## Materials and Methods

### Design

This study comprises a 2 (pre-training, post-training) × 3 (ABM-training away from itch, ABM-training toward itch, sham training: equal allocation ratio) mixed research design with AB to itch pictures, AB to itch words and sensitivity to mechanically induced itch as dependent variables. This study was pre-registered in the Netherlands Trial Registry under number: NL6134 (/NTR6273). The protocol was approved by the Psychology Research Ethics Committee of Leiden University (CEP17-0228/116).

### Participants

Participants were recruited through advertisements on social media, at Leiden University, and via the Leiden University Research Participation system SONA systems Ltd. (Tallinn, Estonia). Recruitment and testing took place between March and May 2017. Inclusion criteria for participation were being aged between 18 and 30 years and being proficient in the Dutch language. Exclusion criteria were current itch or pain ≥3 on a numeric rating scale (NRS) from 0 (no itch/pain) to 10 (worst imaginable itch/pain), diagnosis of a chronic itch or pain condition (e.g., eczema or rheumatoid arthritis), psychiatric diagnosis (e.g., major depression or ADHD), color blindness, dyslexia, and impairment in visual acuity that is not corrected with glasses or contact lenses. All participants provided written informed consent for their participation in the study.

### Procedure

**Figure 1:**
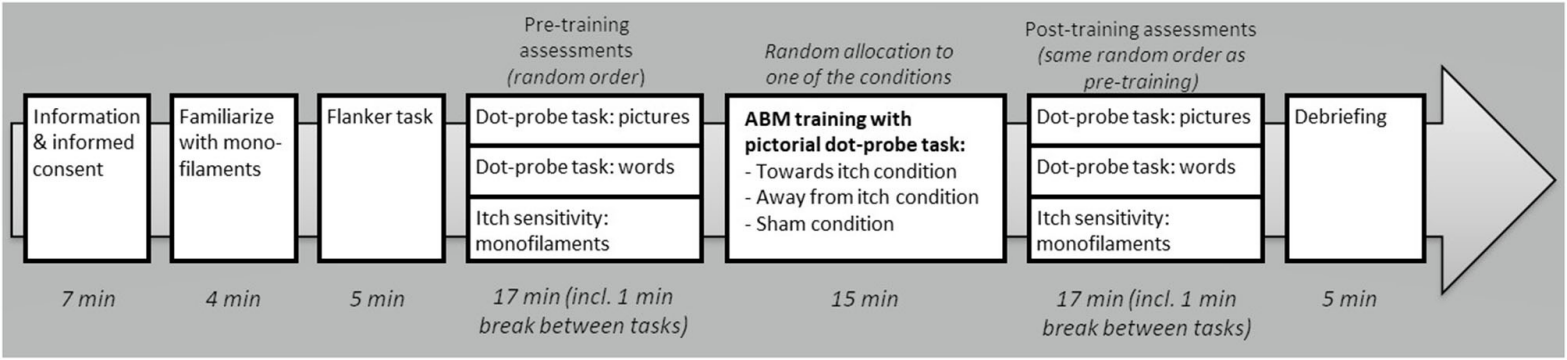
Timeline of the experimental session (total ca. 1 h:10 min).

Potential participants were informed about the study via written information and, when interested in participation, they were asked to fill out (online) self-report questionnaires, which also included questions regarding the in- and exclusion criteria (see section Self-Report Questionnaires). When found eligible for participation, participants were instructed to refrain from intake of alcohol and drugs 24 h before the test session and of caffeinated drinks within 1 h before the session started. Adherence to this guideline was checked in the lab (*n* = 1 missing), resulting in 15 participants who had taken alcohol the preceding 24 h (11 of them drank ≤2 units), 5 participants who had taken caffeinated drinks in the preceding hour (all ≤2 units), and none had used drugs. During each session, two experimenters were present, one conducting the practical tasks (e.g., starting computer tasks and itch stimulus application) and the other one guiding participants through the procedures, mainly by providing instructions. Upon arrival at the Leiden University lab (see Figure 1 for a timeline), participants were verbally informed about the study procedures and told that they were free to terminate the experiment at any time. Next, participants signed the informed consent and rated their current levels of spontaneous itch and pain on the NRSs. Participants were familiarized with the mechanical itch induction by applying Touch test evaluators as described in the section Mechanical Itch Sensitivity (ca. 4 min). Thereafter, the Flanker task (ca. 5 min) was conducted measuring general response inhibition (Flanker task). Consecutively, participants performed the pre-training AB assessments using the dot-probe task with pictures (ca. 5 min), the dot-probe task with words (Dot-Probe Tasks; ca. 5 min), and the mechanical itch induction (ca. 2 min). These tasks were provided in random order, i.e., an independent person had put the randomization information in opaque envelopes stratified by participant's sex and handedness. After these pre-training assessments, participants were randomized to one of the three ABM-training (ca. 15 min) conditions (participants were blind for receiving any intervention). Post-training assessments were carried out in the same order as the pre-training assessments of that specific participant. Upon completion of the tasks, participants were debriefed about the purposes of the study and reimbursed for their participation.

### Measures

All computer tasks were run on a desktop computer with Microsoft Windows 7 attached to a Philips Brilliance 220B TFT screen (Resolution 1,280px × 1,024px, 60 Hz). Both the Dot-probe task and the Flanker task were programmed and run in E-prime 2.0 (Psychology Software Tools Inc., Sharpsburg, PA, USA). Randomization to one of the ABM-training conditions was also done in E-prime based on participant number (this was unknown to the experimenters), with separate lists for males and females. E-prime automatically started the correct condition the participant was randomized to, so the participant was blinded. Responses were given using finger response buttons, one for each hand [Pushbutton Switch, SPDT, Off-(On)] connected to a serial response box model 200A (Psychology Software Tools Inc. Sharpsburg, PA, USA). During the tasks, participants kept their head in a chin rest to keep the distance to the screen at 54 cm.

#### Dot-Probe Tasks

A dot-probe paradigm was used to measure AB toward itch pictures and words. The dot-probe paradigm assumes that being attentive to a stimulus speeds up responding to targets at the focused location (congruent trials) when compared to the opposite location (incongruent trials). There were in total 60 stimulus pairs of an itch-related and a neutral stimulus (40 picture-pairs and 20 word-pairs with Dutch words). Itch stimuli depicted a hand scratching him-/herself on e.g., the head, limb and back. Neutral stimuli depicted objects, e.g., light bulb, doorbell, and spoon. The itch stimuli had been validated earlier [see (38)] on the basis of their applicability to itch (average itch scores per task ranged from 2.7 to 2.8 for the dot-probe tasks assessing AB and the ABM-training task rated on a Likert scale from 1 (not applicable to itch) to 5 (very applicable to itch), non-applicability to pain (average pain scores ranged from 1.0 to 1.4 on a 1 to 5 Likert scale for pain) and slightly negative valence [ranging from −1.2 to −1.1 on a Likert scale ranging from −5 (very negative) to 5 (very positive)], whereas the neutral stimuli were characterized as neither itchy nor painful (average itch and pain scores were 1.1 at maximum), and were of neutral valence (average score ranging from 0.1 to 0.2) (38). Based on the validation scores, the picture-pairs were randomized in advance over three pictorial dot-probe tasks, i.e., two regular dot-probe tasks (10 stimulus-pairs each) and one training dot-probe task (20 stimulus-pairs). The word-pairs were randomized in advance, across two regular dot-probe tasks. Randomization of the stimulus-pairs occurred on basis of the previously acquired validation ratings on itch in order to make sure that the itch ratings would overall be comparable across the dot-probe tasks used. For each participant, the order of the two dot-probe tasks was randomized (i.e., one was administered pre-training and the other post-training). The regular dot-probe tasks contained 40 trials each (14). Half of the trials were congruent (itch stimulus and target at same location) and half of the trials were incongruent (itch stimulus and target at opposite location). The proportion of itch stimuli displayed in the upper and lower half of the screen was equal over all trials. Right before the experimental trials in the pre-training dot-probe tasks only, there were 16 practice trials with neutral-neutral stimulus pairs. Feedback on the accuracy of responses to the targets was included. A trial was constructed as follows: First, a fixation point was shown in the middle of the screen for 500 ms. Upon disappearance, a stimulus pair was presented on the screen for 500 ms (27, 29, 34). One stimulus of each pair was presented centrally at the lower half of the screen (20% height), and the other was presented centrally at the upper half of the screen (80% height). The stimulus-pair was followed by a target stimulus that consisted of two dots aligned either horizontally or vertically. The target stimulus was presented at either one of the stimulus locations until the participant pressed a response button, with a maximum response window of 1,500 ms. Correct response-mapping was counterbalanced across participants, i.e., right button for horizontally oriented target stimuli and left button for vertically oriented target stimuli or vice versa (e.g., a participant responded with the right button to horizontally oriented targets in all dot-probe tasks). Reaction times and accuracy in responding to the targets were measured.

The ABM-training exclusively contained pictorial stimuli. The training task was comparable to the regular dot-probe tasks, but contained 320 trials (24) and the locations of the targets as opposed to the itch pictures was manipulated in the ABM-training conditions that were trained towards and away from itch. Specifically, in the training condition towards itch, 100% of the trials were congruent (i.e., at the itch picture location), whereas in the ABM-training away from itch condition, 100% of the trials were incongruent (i.e., at the neutral picture location). In the sham training condition, 50% of the targets were displayed congruently and 50% were shown incongruently to the itch picture location, akin the regular dot-probe tasks. One minute breaks were built-in after every 40 trials.

#### Mechanical Itch Sensitivity

Sensitivity to touch evoked itch (STI) was assessed, using three von Frey monofilaments (4.08 mN, 4.17 mN, and 4.31 mN; Stoelting, North Coast Medical, Gilroy, CA) as described in previous research (39). The monofilaments were applied to the non-dominant inner forearm. Each filament was applied for 1 s in triplicate after which the participants rated the evoked itch per filament on the NRS for itch.

#### Flanker Task

This task (40, 41) was used to measure general attentional inhibition. In each trial, 5 numbers were shown. The middle number was the target stimulus, which was flanked by non-target stimuli. The flankers could be congruent to the target stimulus, e.g., 44444 or incongruent, e.g., 44244. Half of the trials were congruent and half were incongruent and the use of 2's and 4's was balanced across the task. The task contained 120 experimental trials and 8 practice trials without feedback at the beginning of the task. The left response button was used to indicate “2” as target and the right button was used to indicate “4.” A short break was included half-way the task if desired. The average reaction time to the congruent vs. the incongruent target stimuli is the outcome measure.

#### Self-Report Questionnaires

Questions assessing demographic information (e.g., age) and information required to screen for in- and exclusion criteria (e.g., having medical or psychiatric conditions, experiencing spontaneous itch or pain) were included. In addition, *attentional focus on bodily sensations* was measured using both the Body Vigilance Scale (BVS with Cronbach alpha 0.70) as previously described (i.e., only including the sub items of question 4 that concern bodily sensations) (11) with two additional items assessing one's attention directed toward itch and pain and the Pain Vigilance and Awareness Questionnaire adjusted for itch (PVAQ-I with Cronbach alpha 0.88). Adjustments for itch were made by substituting the word “pain” by “itch” for all concerning items. This procedure was also applied to the following questionnaires originating from the pain field. *Catastrophizing* about itch was measured using the Pain Catastrophizing Scale adjusted for itch (PCS-I with Cronbach alpha 0.91) (39). *Experience of Cognitive Intrusion of itch* was assessed using the Experience of Cognitive Intrusion of pain scale adjusted for itch and, accidentally, with scales ranging from 1 to 6 for each item instead of 0 to 6 like the original version (ECIP-I with Cronbach alpha 0.96). *Attentional disengagement from itch and pain* was assessed using two Likert scales ranging from 1 (not at all able to disengage attention) to 5 (always able to disengage attention) (12). Finally, *Neuroticism* was measured with the Eysenck Personality Questionnaire revised short scale (EPQ-RSS with Cronbach alpha = 0.72) (42). All self-report questionnaires were administered in Dutch using the online system Qualtrics (Provo, Utah, USA).

#### Statistical Analyses

All statistical analyses were conducted using SPSS 25.0 software (IBM SPSS Statistics for Windows, Armonk, NY, USA) if not specified otherwise. All values displayed are means ± standard deviation (SD), if not stated otherwise. Effect sizes were reported as partial eta squared ($\eta_{\text{p}}^{2}$). A *p* < 0.05 was considered statistically significant.

For the dot-probe tasks, reaction times (RT) were extracted from E-prime for trials with RT ≥ 150 ms (0.2 and 0.08% of the RT were excluded for the picture and word tasks, respectively) and trials with correct responses (7.2 and 8.5% of the RT were excluded from the picture and word tasks, respectively). Cases for which no responses were recorded due to a programming error (see section Sample) were excluded from the respective analysis. Participants' data that had accuracy levels below 70% were excluded from the respective analyses; in the case that <70% of the trials in the training were incorrect, this participant was not included in any of the analyses. For the pre- and post-training dot-probe tasks, AB-indices were calculated by subtracting the RT of the congruent trials from the RT of the incongruent trials (RT_incongruent_-RT_congruent_). A positive AB-index indicates an AB toward itch, whilst a negative AB-index indicates an AB away from itch. All variables to be included in the statistical analyses were checked for normality.

First, baseline between-group differences in demographics, current spontaneous itch and pain, total scores of the questionnaires, AB toward itch pictures and words, mechanically evoked itch, and general attentional inhibition (i.e., Flanker task) were assessed using Kruskal-Wallis-tests for interval variables (due to a violation of normality for most variables) and a Chi-square-test for the dichotomous variable sex. Second, the presence of ABs toward itch pictures and words at baseline was tested using one sample *t*-tests checking whether the AB-index significantly differed from 0. Additionally, effectiveness of participants' attentional inhibition (entire group) was checked by comparing the RT on the congruent and incongruent trials of the Flanker task as within-subjects factor in a repeated measures analysis of variance (RM-ANOVA).

For the primary aim to assess whether ABM-training resulted in altered attention to itch pictures, a 2 × 2 × 3 RM-ANOVA was conducted with the within-factors *itch congruency* (congruent vs. incongruent trials) and *time* (pre- vs. post-training assessments) and the between-factor *condition* (ABM-training away, ABM-training towards, sham training). Additionally, *post-hoc* moderation analyses were carried out using the Process Macro v3.3 (43) (model 1) in SPSS to investigate whether the effects of the training were different depending on the baseline level of AB toward the main outcome of itch pictures. Here, *condition* was the independent variable (X), the training effect on itch pictures (AB-index_post−training_-AB-index_pre−training_) was the dependent variable (Y; centered) and the pre-training AB towards itch pictures was the moderator variable. Another *post-hoc* RM-ANOVA tested the change in AB-index for the itch pictures before vs. after the training in the entire sample.

For the secondary aim to assess the effect of the ABM-training on itch words, a RM-ANOVA akin the one with pictures was conducted with the RT of the word dot-probe tasks. For the mechanical itch sensitivity outcome, pre- and post-training levels of evoked itch, as subjectively rated on NRS, were compared using a RM-ANOVA with the within-factor *time* (mechanically evoked itch pre- vs. post-training) and the between-factor *condition* (ABM-training away, ABM-training towards, sham training). Finally, to test associations between the main study outcomes and the individual characteristics, Spearman correlation coefficients (ρ) were calculated for each condition separately. Specifically, the ABM-training effects on the AB toward itch pictures and AB toward itch words (both AB-index post-training–AB-index pre-training) as well as itch sensitivity (pre–post assessment) were correlated with both the Flanker index (RT_incongruent_-RT_congruent_) and the total scores of the self-report questionnaires. The Sidak-Holm correction was applied for all RM-ANOVAs when performing *post-hoc* tests.

Reliability of the dot-probe tasks was assessed with split-half reliabilities. These were calculated with R (R version 4.0.3) (44) with the “splithalfr” package (45). Reliability of the AB index was calculated for all four versions of the dot-probe task (version 1 and version 2 with pictures as well as version 1 and version 2 with words) of all participants that were included in the analyses. The function used a Monte-Carlo splitting technique to estimate 5,000 split-half samples that were used to estimate Spearman-Brown correlations for all 5,000 samples. The resulting mean and median coefficients of all 5,000 samples accompanied by the minimum, maximum, and interquartile range were calculated per task.

## Results

### Sample

We aimed to include 40 participants per condition as this would be sufficient to detect a small effect (in GPower 3.1.6, effect size f of 0.10; with an alpha of 0.05, power of 0.80 and an estimated correlation between the pre- and post- measurements between 0.75 and 0.80). On top, 5% participants extra were tested in order to be able to overcome potential data loss. Therefore, 126 participants had been included in the study. For the following reasons, data of several participants could not be used in data analyses: Seven participants responded differently to the orientation of the dots during the training task as opposed to the pre- and post-training dot-probe tasks due to incorrectly provided instructions (e.g., they were instructed to respond to horizontal oriented targets with the right button during the pre- and post-training dot-probe tasks and with the left button during training). Due to a programming error, data of the dot-probe picture and word tasks had not been recorded /could not be retrieved for two participants and for similar reasons data of another 12 participants was unavailable for the word tasks (amongst them, there was one participant of whom data of the mechanically evoked itch were missing, too). Moreover, one participant was excluded from the main analysis because of exceeding the pre-determined 30% error rate (specifically 33% errors) for the post-training dot-probe picture task. None of the participants had to be excluded based on their number of errors during the ABM-training; at maximum 18% of the trials were incorrect (*n* = 1). Finally, 116 participants could be included in the main analysis with the pictorial stimuli, 105 in the secondary analyses with the word stimuli, and 116 in the analyses for mechanically evoked itch. The sample of 116 participants was mostly female (74%), right-handed (89%) and most participants were following or had finished tertiary education (85%). Participants' baseline characteristics did not differ across training conditions (Table 1). Median levels of spontaneous itch and pain at baseline were 0.0.

**Table 1:** Baseline individual characteristics per attention bias modification (ABM) training condition.

	**Total sample**	**ABM-training away from itch (*n* = 38)**	**ABM-training towards itch (*n* = 40)**	**Sham training (*n* = 38)**	**Statistic for condition difference**
**Prior to session**					
Sex (*n* M/F)	30/86	11/27	10/30	9/29	$\chi_{\left(2\right)}^{2}$ = 0.298, *p* = 0.861
Age	22 (21; 23)	22 (21; 23)	22 (21; 23)	21 (21; 23)	*H*_(2)_ = 0.021, *p* = 0.990
Body vigilance (BVS)	3.1 (2.1; 4.2)	3.2 (1.6; 4.0)	2.9 (1.8; 3.8)	3.1 (1.9; 3.9)	*H*_(2)_ = 0.044, *p* = 0.978
Single item for attentional focus on itch/pain (0–10)	1.7 (0.3; 3.8)/2.5 (0.3; 4.7)	1.3 (0.2; 3.9)/2.2 (1.5; 5.0)	1.6 (0.7; 3.2)/2.9 (0.6; 5.0)	1.7 (0.1; 3.8)/1.8 (0.5; 5.0)	*H*_(2)_ = 0.418, *p* = 0.811/*H*_(2)_ = 0.910, *p* = 0.635
Itch vigilance and awareness (PVAQ-I)	25.0 (17.5; 32.5)	24.5 (17.8; 31.0)	22.5 (15.3; 32.0)	28.0 (16.8; 32.8)	*H*_(2)_ = 1.067, *p* = 0.587
Single item for attentional disengagement from itch/pain	4.0 (3.0; 5.0)/4.0 (3.0; 4.0)	4.0 (3.0; 5.0)/4.0 (3.0; 4.0)	4.0 (3.0; 5.0)/4.0 (3.0; 4.0)	5.0 (3.8; 5.0)/4.0 (3.0; 5.0)	*H*_(2)_ = 3.883, *p* = 0.143/*H*_(2)_ = 2.770, *p* = 0.250
Itch catastrophizing (PCS-I)	6.0 (1.0; 11.0)	7.5 (3.5; 12.0)	6.0 (2.0; 12.8)	5.5 (2.0; 12.3)	*H*_(2)_ = 0.454, *p* = 0.797
Cognitive intrusions of itch (ECIP-I)	13.5 (8.0; 19)	14.0 (11.0; 22.0)	15.0 (11.0; 22.3)	13.0 (11.0; 22.3)	*H*_(2)_ = 0.372, *p* = 0.830
Neuroticism (EPQ-RSS)	3.0 (2.0; 5.0)	3.0 (2.0; 5.0)	3.5 (2.0; 6.0)	3.0 (1.0; 6.0)	*H*_(2)_ = 1.806, *p* = 0.405
**During session**					
General response inhibition (Flanker index)	51.0 (37.3; 64.7)	52.9 (40.8; 66.0)	54.7 (39.4; 64.1)	47.2 (31.8; 60.8)	*H*_(2)_ = 1.820, *p* = 0.403

*Values displayed are medians (interquartile range: IQR) and absolute numbers for the variable sex*.

*EPQ-RSS, Eysenck Personality Questionnaire revised short scale (theoretical range 0–12 neuroticism subscale); Single items assessing attentional focusing on itch and pain (theoretical range 0–10); BVS, Body Vigilance Scale (theoretical range 0–10); PVAQ-I, Pain Vigilance and Awareness Scale, adjusted for itch (theoretical range 0–80); PCS-I, Pain Catastrophizing Scale, adjusted for itch (theoretical range 0–52); ECIP-I, Experience of Cognitive Intrusions of Pain, adjusted for itch (theoretical range original version 0–60—in the current study 10–60); Single items about attentional disengagement (theoretical range 1–5). The flanker index was calculated by subtracting the RT for congruent trials from the RT from incongruent trials. Because most questionnaires were not-normally distributed, the conditions were compared using non-parametric Kruskal-Wallis-tests (H) on most outcomes, except for sex, which was compared in a Chi-square-test*.

### Dot-Probe Tasks

Reliability was good for all versions of the task with a mean Spearman-Brown coefficient between 0.61 and 0.71, based on 5,000 split-half samples, see Table 2. For the dot-probe task with pictures and the itch sensitivity analyses, one outlier (>3 interquartile range) was excluded (final *n* = 115 for both outcomes). Variables for the dot-probe task with pictures were log-transformed to obtain normal distribution.

**Table 2:** Reliability coefficients for the different versions of the dot-probe tasks.

		**Mean (range)**	**Median (IQR)**
Dot-probe tasks with pictures	Version 1	0.68 (0.34–0.84)	0.68 (0.64; 0.72)
	Version 2	0.71 (0.41–0.70)	0.72 (0.68; 0.75)
Dot-probe tasks with words	Version 1	0.67 (0.23–0.86)	0.68 (0.62; 0.72)
	Version 2	0.60 (0.20–0.84)	0.61 (0.55; 0.66)

*Mean and the range of the Spearman-Brown coefficient of 5,000 split-half samples are reported, as well as the median and interquartile range (IQR)*.

#### Pre-training AB Toward Itch Pictures and Words

The one-sample *t*-test with the pre-training AB-index differed significantly from zero [*t*_(114)_ = −2.26, *p* = 0.026], indicating that participants overall were focused away from the itch pictures at baseline (see **Table 3** for descriptive values). There was no pre-training AB for itch words [*t*_(104)_ = 0.248, *p* = 0.805] (**Table 4**). The RM-ANOVA demonstrated that ABM-training conditions did not significantly differ in their pre-training AB-index for itch pictures [*F*_(2,113)_ = 0.09, *p* = 0.911, *η*_*p*_^2^ = 0.002] or words [*F*_(2,102)_ = 0.559, *p* = 0.574, *η*_*p*_^2^ = 0.011].

#### Training AB Toward Itch Pictures

**Figure 2:**
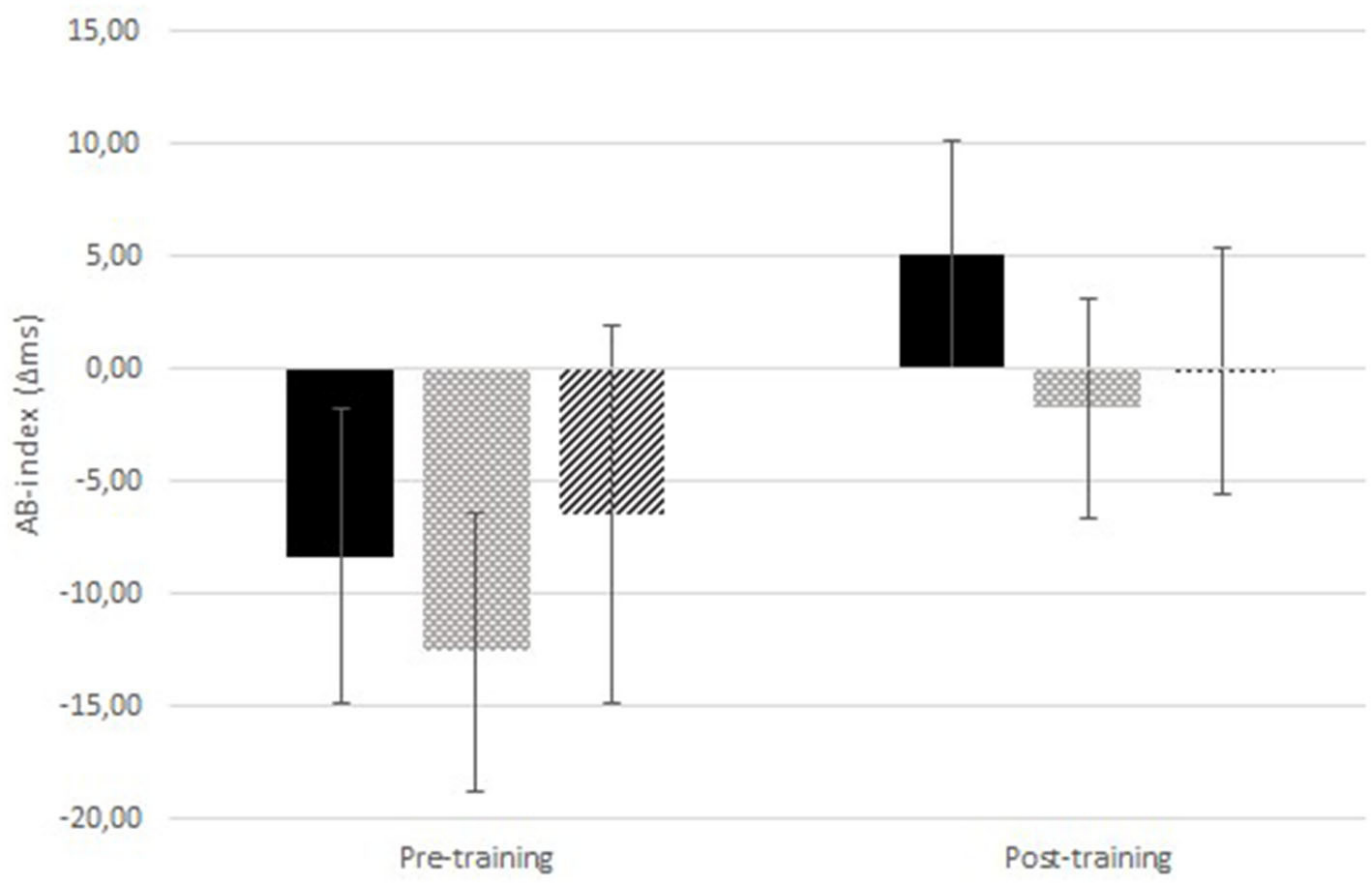
Attentional Bias (AB)-index for the itch pictures pre- and post-ABM-training. Results are displayed for the ABM-training away from itch (black; *n* = 38), ABM-training toward itch (light gray dots; *n* = 40), and the sham training (intermediate gray stripes; *n* = 37). Positive values indicate an AB toward itch. Error bars represent standard errors of the mean.

The 2 (time: pre- vs. post- training) × 2 (itch congruency: itch-congruent vs. itch-incongruent) × 3 (training condition) RM-ANOVA, testing the main hypothesis whether training attention away and towards pictorial itch stimuli altered attention toward itch pictures (Figure 2 and Tables 3A,B) showed no significant time × itch congruency ×condition effect [*F*_(2,112)_ = 0.41, *p* = 0.663, *η*_*p*_^2^ = 0.007]. There was a significant main effect of time [*F*_(1,112)_ = 199.87, *p* < 0.001, *η*_*p*_^2^ = 0.641], showing that RT were shorter after the training than before (Tables 3A,B). There was neither a significant main effect of congruency [*F*_(1,112)_ = 2.46, *p* = 0.120, $\eta_{\text{p}}^{2}$ = 0.022] nor of condition [*F*_(2,112)_ = 0.753, *p* = 0.473, $\eta_{\text{p}}^{2}$ = 0.013].

**Table 3A:** Mean ± standard deviation of reaction times for the trials congruent and incongruent to the itch pictures of the dot-probe tasks administered pre- and post-attention bias modification (ABM)-training, displayed for the total sample and per training condition.

		**Total sample (*n* = 115)**	**ABM-training away from itch (*n* = 38)**	**ABM-training towards itch (*n* = 40)**	**Sham training (*n* = 37)**
Pre-training	Congruent trials	545.4 ± 77.5	544.7 ± 67.8	537.1 ± 75.2	555.0 ± 89.5
	Incongruent trials	536.2 ± 77.8	532.1 ± 65.8	528.7 ± 83.8	548.5 ± 83.0
	AB-index	−9.1 ± 43.4	−12.6 ± 38.0	−8.4 ± 41.5	−6.5 ± 51.0
Post-training	Congruent trials	474.0 ± 63.3	475.1 ± 62.0	462.7 ± 50.2	485.0 ± 75.8
	Incongruent trials	475.1 ± 68.9	473.4 ± 65.4	467.8 ± 58.7	484.9 ± 82.1
	AB-index	1.2 ± 31.5	−1.7 ± 30.0	5.1 ± 31.8	−0.1 ± 33.2

**Table 3B:** Median (and interquartile range; IQR) of reaction times for the trials congruent and incongruent to the itch pictures of the dot-probe tasks administered pre- and post-attention bias modification (ABM)-training, displayed for the total sample, and per training condition.

	**Trial type**	**Total sample (*n* = 115)**	**ABM-training away from itch (*n* = 38)**	**ABM-training towards itch (*n* = 40)**	**Sham training (*n* = 37)**
Pre-training	Congruent	534.2 (488.9; 580.7)	535.0 (492.7; 579.7)	529.3 (485.5; 567.1)	537.4 (491.3; 605.4)
	Incongruent	521.3 (473.0; 579.8)	515.6 (480.2; 574.2)	511.1 (468.6; 566.5)	531.6 (476.0; 613.0)
Post-training	Congruent	464.3 (429.0; 507.5)	453.7 (430.2; 510.5)	458.8 (426.9; 501.8)	480.2 (424.5; 536.1)
	Incongruent	463.2 (426.4; 516.6)	460.6 (423.2; 513.6)	462.6 (424.4; 503.6)	480.9 (430.3; 533.6)

#### Training AB Toward Itch Words

The 2 × 2 × 3 RM-ANOVA testing the secondary hypothesis whether ABM-training away and towards pictorial itch stimuli would generalize to changes in AB toward itch words showed no significant time x itch congruency x condition effect [*F*_(2,102)_ = 0.091, *p* = 0.913, $\eta_{\text{p}}^{2}$ = 0.002]. The significant main effect of time [*F*_(1,102)_ = 118.29, *p* < 0.0001, $\eta_{\text{p}}^{2}$ = 0.537] showed RT to be shorter after the training than before (Table 4). No significant main effect of itch congruency [*F*_(1,102)_ = 0.194, *p* = 0.661, $\eta_{\text{p}}^{2}$ = 0.002], but a significant main effect of condition was found [*F*_(2,102)_ = 5.842, *p* = 0.004, $\eta_{\text{p}}^{2}$ = 0.103] with contrasts showing that RT for targets was faster in both, the condition trained away and towards itch, than in the sham training condition [mean difference (*MD*) = −39.4, standard error (*SE*) = 15.2, *p* = 0.032, and *MD* = −47.7, *SE* = 14.9, *p* = 0.005, respectively].

**Table 4:** Mean ± standard deviation of reaction times for the trials congruent and incongruent to the itch words of the dot-probe tasks administered pre- and post-training, displayed for the total sample and per attention bias modification (ABM)-training condition.

		**Total sample (*n* = 105)**	**ABM-training away from itch (*n* = 33)**	**ABM-training towards itch (*n* = 36)**	**Sham training (*n* = 36)**
Pre-training	Congruent trials	557.6 ± 84.9	544.6 ± 87.9	535.9 ± 67.4	591.1 ± 89.4
	Incongruent trials	558.5 ± 85.0	550.2 ± 90.7	537.9 ± 69.5	586.8 ± 88.2
	AB-index	0.9 ± 39.2	5.5 ± 35.9	2.0 ± 32.0	−4.3 ± 48.1
Post-training	Congruent trials	497.6 ± 62.9	483.1 ± 47.6	481.6 ± 62.4	527.0 ± 66.5
	Incongruent trials	494.1 ± 61.3	487.3 ± 55.4	476.7 ± 55.3	517.8 ± 66.2
	AB-index	−3.5 ± 36.0	4.2 ± 34.9	−4.9 ± 38.1	−9.2 ± 34.7

### Itch Sensitivity

On the pre-training assessment of mechanically evoked itch (log-transformed), training conditions did not significantly differ [*F*_(2,112)_ = 0.458, *p* = 0.634, $\eta_{\text{p}}^{2}$ = 0.008]. The 2 × 3 RM-ANOVA testing the secondary hypothesis whether ABM-training attention away and towards pictorial itch stimuli would generalize to changes in itch sensitivity (residuals were normally distributed after excluding the outlier, so variables were not transformed) obtained no significant time × condition effect [*F*_(2,112)_ = 0.259, *p* = 0.772, $\eta_{\text{p}}^{2}$ = 0.005]. There was neither a significant main effect of time [*F*_(1,112)_ = 0.294, *p* = 0.588, $\eta_{\text{p}}^{2}$ = 0.003] nor of condition [*F*_(2,112)_ = 0.625, *p* = 0.537, $\eta_{\text{p}}^{2}$ = 0.011]. See Table 5 for descriptive values.

**Table 5:** Mean ± standard deviation of mechanically evoked itch measured on a numeric rating scale from 0 (no itch) to 10 (worst itch imaginable) displayed for the total sample and per attention bias modification (ABM) training condition.

	**Total sample (*n* = 115)**	**ABM-training away from itch (*n* = 37)**	**ABM-training toward itch (*n* = 39)**	**Sham training (*n* = 39)**
Pre-training itch^a^	1.8 ± 1.5	2.0 ± 1.5	1.7 ± 1.4	1.8 ± 1.5
Post-training itch	1.8 ± 1.5	2.0 ± 1.8	1.6 ± 1.4	1.9 ± 1.5

a *For the pre-training analysis, the variables were not-normally distributed, hence the medians and interquartile ranges are reported here. Median (IQR) was for the total sample 1.5 (0.7; 2.7), for the ABM-training away from itch 1.7 (0.9; 2.8), for the ABM-training toward itch 1.3 (0.5; 2.8), and for the sham training 1.7 (0.5; 2.7)*.

### *Post-hoc* Analyses

*Post-hoc* moderation analysis showed that the effect of the ABM-training on AB for itch pictures was not moderated by the pre-training level of AB for itch pictures (Table 6). Additionally, over the entire sample, AB for itch pictures increased significantly [*F*_(1,114)_ = 5.16, *p* = 0.025, $\eta_{\text{p}}^{2}$ = 0.043].

**Table 6:** Linear model of pre-training attention bias (AB)-index for itch pictures as predictor (moderator) of the attention bias modification (ABM) training effect [*n* = 115; 95% confidence intervals (CI) and standard errors based on 1,000 bootstrap samples].

	***B* (CI)**	**SEB**	***t***	***p***
Constant	−10.40 (−16.21, −4.60)	2.93	−3.551	0.001
Condition	2.56 (−4.88, 10.01)	3.76	0.683	0.496
Pre-training AB-index for itch pictures (Centered)	0.85 (0.71, 0.98)	0.07	12.516	<0.001
Pre-training AB-index for itch pictures X Condition effect	0.12 (−0.04, 0.28)	0.08	1.478	0.142

*R^2^ = 0.61. The training effect is defined by the change in AB-index before minus after the training (a positive value indicates a decreased AB after training)*.

### Flanker Effect

A significant Flanker effect [*F*_(1,115)_ = 419.76, *p* < 0.001, $\eta_{\text{p}}^{2}$ = 0.785], with faster RT for congruent (423.8 ± 78.7) than incongruent trials (472.2 ± 69.6) indicated attentional inhibition across the sample.

### Associations With Individual Characteristics

Of all Spearman correlation coefficients between the individual characteristics and the ABM-training effect on the different outcomes, only a significant correlation was found between high levels of neuroticism and larger increases in mechanically induced itch in the ABM-training condition towards itch (ρ_*S*_ = 0.35, *p* = 0.03). Another significant correlation emerged in the sham training condition, which was between a better disengagement ability from itch and a larger decrease in mechanically induced itch (ρ_*S*_ = 0.46, *p* = 0.004).

## Discussion

We assessed the effects of attention bias modification (ABM)-training on healthy individuals' attentional bias (AB) toward itch pictures and itch words as well as on sensitivity to mild itch. This is the first proof-of-principle ABM-training study in the field of itch. Specifically, we also included a condition in which attention was trained toward itch, besides the training away from itch and a sham condition. In contrast to expectations, ABM-training did not alter attention to itch pictures. Furthermore, ABM-training using itch pictures did not affect AB toward itch words nor itch sensitivity. Additionally, of the individual characteristics, only neuroticism was associated with a larger training effect, specifically with an increase in mechanically evoked itch in the condition trained towards itch. In sum, although we expected ABM-training to be promising for itch, given the contagiousness and attention-capturing characteristics of itch (6, 13, 15), we can conclude that the hypotheses could not be confirmed.

Given the novelty of an ABM-training for itch, comparing current findings with findings of previous ABM-trainings for pain may provide some further insight. Largely inspired upon previous ABM research on pain (27, 29, 34), we opted for a 500-ms stimulus display time, the use of pictures, and a target discrimination instead of a localization task. Yet, we can conclude that although results are not in line with our hypotheses, current findings are also not completely unexpected when inspecting the ABM-training literature. Indeed, although initial results of ABM for pain-related information showed promising results (26, 27), more recent studies indicate that ABM-trainings for pain are ineffective in changing AB toward pain in healthy participants (28, 29, 35). For both, potential moderation of ABM-training effects by baseline levels of AB for itch and generalization to another type of AB (i.e., from pictures to words), only preliminary evidence from the pain literature is available (37). Moreover, generalization occurred only from words to pictures and not vice versa (27). That itch sensitivity was unaffected by the ABM-training is also partly in line with previous pain studies. Specifically, some studies favored effectiveness of ABM-training on experienced pain or pain thresholds (26, 27, 29), while others did not find effects on pain outcomes or only for some pain outcomes (28, 29, 35). Furthermore, in multiple studies changes in somatosensory pain outcomes were not accompanied by changes in AB for pain (27, 29). Comparable mixed results emerged in other fields, such as anxiety for which ABM-trainings were originally developed (21, 46). Overall, previous findings of the effects of ABM-training are mixed or preliminary.

Various explanations of current findings in relation to the inconsistent evidence for ABM-training studies for pain [see also (35)] can be considered. First, the present study included a sham training to inform about potential distinct effects of each training condition. Nonetheless, previous pain research often compared an ABM-training toward pain with an ABM-training away from pain, which likely obtains larger effects due to comparison of the most “extreme” conditions. Noteworthy, *post-hoc* analyses comparing our extreme conditions does not change the conclusions. Second, lack of effectiveness on AB for pictures and words may relate to the fact that after the active training conditions (including either congruent or incongruent trials), both congruent and incongruent trials were offered in the dot-probe tasks to assess AB for itch. This may have diluted potential training effects. Moreover, given the null-findings of an AB towards itch pictures, the lack of a generalization towards the itch words and sensitivity is not surprising. Third, participants did not have a baseline AB for itch stimuli, as would be expected (14, 17). This generally hampers the possibility to train attention away, although also no moderation by the baseline AB levels was found. Moreover, this does not explain the lack of training effects for those trained towards itch, particularly because at baseline average RT pointed in the opposite direction, which could be interpreted as attentional avoidance of itch pictures. Nevertheless, previous ABM-trainings away from pain have shown to be effective in reducing pain outcomes despite the absence of a baseline AB towards pain (26, 28, 29). However, the current study did not find effects on itch sensitivity either. This does not seem to be due to the levels of itch induced, which were comparably moderate in previous studies (39, 47), in which itch reduction was effectuated (by heterotopic stimulation) (39). Fourth, as elaborated on by Wiers et al. (36) a proof-of-principle study in healthy individuals entails that participants are not aware of receiving an intervention and have no motivation to change responses. Motivation to pursue certain goals, e.g., getting rid of itch, as well as having positive expectations about an intervention play an important role in the experience and treatment of various symptoms (13, 32, 48–50). Therefore, possible effects to be obtained are probably smaller in healthy individuals than in patients.

Interestingly, at baseline, participants were faster on itch incongruent than congruent trials for the itch pictures [also seen in (16); this may be related to the picture content, e.g., the itch pictures are of weak emotional valence (50) to the healthy individuals], which could be indicative of attentional avoidance of itch. This “avoidance bias” hampered the ability to train attention away, and simultaneously increased the opportunity to train attention towards itch. In fact, the “avoidance bias” was abolished, as demonstrated by the lack of a significant itch-congruency effect after the training irrespective of the condition participants were in (though seemingly mostly in the training towards itch; Figure 2). This unexpected finding of increased attention to itch in the entire sample is in the direction opposite to what is desirable. This may have been caused by participants becoming generally more familiar with the picture content over time. Additionally, particularly in the pre-training assessment, the stimuli were new to the participants and the neutral pictures apparently drew more attention than the itch pictures. This may be related to the more heterogeneous content of the neutral (various objects) than the itch pictures (scratching hand), making the neutral pictures more novel (51). It may be worthwhile to explore if the attention increase to the itch pictures would still occur when presenting stimuli subliminally. Noteworthy, participants' responses were significantly faster after the training than before, which can be attributed to a task learning effect.

Several limitations and directions for future research should be mentioned. First, although reliability of the dot-probe tasks in the current study was adequate, generally the use of dot-probe tasks to measure AB (not so much to train attention) has recently been questioned because attention may vary highly across trials which is not reflected by the calculated average reaction times (50, 52). However, the majority of, if not all, ABM-trainings used the dot-probe paradigm with comparable analyses, and some were successful. Nevertheless, future studies may benefit from using other tasks, e.g., the dual probe task variant (53), as well as eye-tracking methodology to fully capture the fluctuating process of attention over time (50, 54). Second, training effects could be assessed on more intense itch stimuli, e.g., cowhage (55). Third, including somatosensory itch stimuli as opposed to visual stimuli in the task would enhance ecological validity. However, because of the lack of spatial attention allocation effects toward somatosensory itch (12, 14, 16), translating the ABM-training paradigm into a somatosensory variant remains challenging. Fourth, current ABM-trainings may be improved by incorporating motivational components, e.g., by implementing reward, gamification, or creating a more representable context (32, 35). It is also worthwhile to explore how to extend and personalize cognitive bias trainings for itch in line with the innovative, promising, theory-driven ABC-training for addiction (49). Actually, the itch-scratch cycle behavior and addiction share common neurobiological mechanisms (56). Finally, when ABM-training for itch would eventually be successful, future studies should also include patients with chronic itch, who are generally motivated to diminish the itch, hence have a baseline AB toward itch that can be targeted [e.g., (50, 57, 58) for results in related fields].

## Data Availability Statement

The raw data supporting the conclusions of this article will be made available by the authors, without undue reservation.

## Ethics Statement

The studies involving human participants were reviewed and approved by Psychology Research Ethics Committee of Leiden University. The patients/participants provided their written informed consent to participate in this study.

## Author Contributions

AvL, DvR, SVD, GC, and RW designed the study and interpreted the data. AL lead the data acquisition, conducted the analyses, and wrote the initial draft. JB supported the conduction of analyses. AvL and JB revised the manuscript according to the critical feedback of DvR, SD, GC, and RW. All authors provided approval for publication of the content and agreed to be accountable for all aspects of the work in ensuring that questions related to the accuracy or integrity of any part of the work are appropriately investigated and resolved.

## Conflict of Interest

The authors declare that the research was conducted in the absence of any commercial or financial relationships that could be construed as a potential conflict of interest.
